# Age reprogramming: cell rejuvenation by partial reprogramming

**DOI:** 10.1242/dev.200755

**Published:** 2022-11-16

**Authors:** Prim B. Singh, Assem Zhakupova

**Affiliations:** Department of Medicine, Nazarbayev University School of Medicine, 5/1 Kerei Zhanibek Khandar Street, Astana 010000, Republic of Kazakhstan

**Keywords:** Age reprogramming, Cellular identity, Epigenetic rejuvenation, H3K9me3, OSKM, Partial reprogramming

## Abstract

‘Age reprogramming’ refers to the process by which the molecular and cellular pathways of a cell that are subject to age-related decline are rejuvenated without passage through an embryonic stage. This process differs from the rejuvenation observed in differentiated derivatives of induced pluripotent stem cells, which involves passage through an embryonic stage and loss of cellular identity. Accordingly, the study of age reprogramming can provide an understanding of how ageing can be reversed while retaining cellular identity and the specialised function(s) of a cell, which will be of benefit to regenerative medicine. Here, we highlight recent work that has provided a more nuanced understanding of age reprogramming and point to some open questions in the field that might be explored in the future.

## Teaser

**Summary:** This Spotlight evaluates models of age reprogramming in light of recent studies that have provided molecular insights into the concept of age reprogramming.

## Introduction

**Fig. 1.**
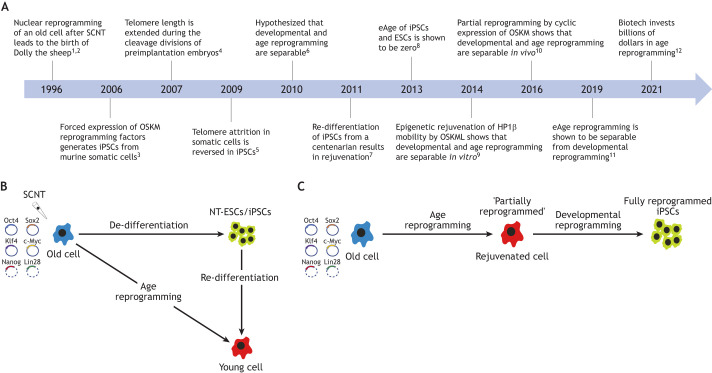
**Age reprogramming.** (A) Key events in age reprogramming research. Numbered references: (1) Wilmut et al., 1997; (2) Sinclair et al., 2016; (3) Takahashi and Yamanaka, 2006; (4) Liu et al., 2007; (5) Marion et al., 2009; (6) Singh and Zacouto, 2010; (7) Lapasset et al., 2011; (8) Horvath, 2013; (9) Manukyan and Singh, 2014; (10) Ocampo et al., 2016; (11) Olova et al., 2019; (12) de Magalhães and Ocampo, 2022. (B) Age reprogramming as a separable element of nuclear reprogramming. Nuclear reprogramming of an old cell (blue) after introduction of the Oct4, Sox2, Klf4 and c-Myc (OSKM) ‘reprogramming factors’ or by somatic cell nuclear transfer (SCNT) results in de-differentiation to an ESC-like state producing iPSCs and nuclear transfer-derived ESCs (NT-ESCs), respectively (green). Re-differentiation of iPSCs and NT-ESCs results in differentiated cells that are rejuvenated (young; red). Age reprogramming aims to bypass the de-/re-differentiation cycle, retaining the specialised functions of the old cell and simply making it younger. Based on Singh and Zacouto (2010). (C) Partial reprogramming as an experimental approach to show that age and developmental reprogramming are separable. ‘Reprogramming factors’ are introduced into an old cell (blue), which is characterised by the expression of age-related markers. During the trajectory from old cell to iPSC (green), a putative stage exists during which the expression of age-related markers is reduced or lost, indicating rejuvenation (red), while the ‘partially reprogrammed’ cell still possesses its specialised characteristics, i.e. it does not exhibit characteristics of embryonic cells. Based on Singh and Zacouto (2010).

Work on animal cloning (Gurdon, 1962; Wilmut et al., 1997) and the induction of induced pluripotent stem cells (iPSCs) using the ‘reprogramming factors’ Oct4, Sox2, Klf4 and c-Myc (OSKM; Dimos et al., 2008; Takahashi and Yamanaka, 2006) has shown that old differentiated cells are able to re-acquire developmental potential via the process of nuclear reprogramming (Fig. 1A). More recently, it was hypothesised that the mechanisms by which an old cell re-acquires developmental potential (developmental reprogramming) are separable from those that reset its age (age reprogramming) (Singh and Zacouto, 2010; Fig. 1A,B). In line with this, experimental evidence indicates that the ‘partial reprogramming’ (Fig. 1C) of old cells after the introduction of reprogramming factors can disentangle age and developmental reprogramming *in vitro* (Manukyan and Singh, 2014) and *in vivo* (Ocampo et al., 2016). Although partial reprogramming has mostly been used for other applications – to enhance tissue regeneration after injury (Chen et al., 2021; Hishida et al., 2022; Wang et al., 2021) and to promote transdifferentiation of one differentiated cell type to another (Maza et al., 2015) – these experiments highlight that age and developmental reprogramming are indeed separable elements of nuclear reprogramming (Fig. 1B,C).

Here, we critically evaluate early models of age reprogramming in light of recent studies (Alle et al., 2022; Browder et al., 2022; Cheng et al., 2022; Chondronasiou et al., 2022; Gill et al., 2022; Lu et al., 2020; Rodríguez-Matellán et al., 2020; Roux et al., 2022; Sarkar et al., 2020; Shahini et al., 2021). We discuss how these studies (detailed in Table S1) provide insight into the concept of age reprogramming and highlight how they are helping to address key questions in the field.

## To what extent is transient de-differentiation required for age reprogramming?

**Fig. 2.**
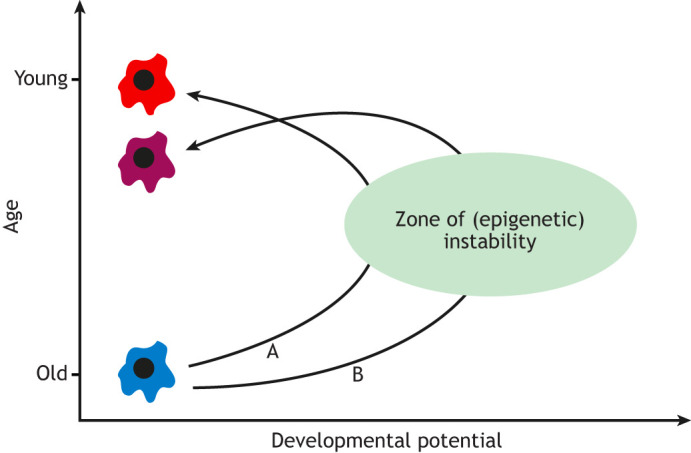
**Transient de-differentiation and age reprogramming.** A **‘**burst’ of OSKM expression results in transient de-differentiation that takes an old, specialised cell (blue) through a zone of (epigenetic) instability (green ellipse). Entry into the zone partially reprogrammes the cell, transiently de-differentiating and briefly expanding its developmental potential (*x*-axis). When OSKM expression is stopped, the partially reprogrammed intermediate returns to its specialised phenotype, with the cell now age reprogrammed and exhibiting a younger ‘age’ (*y*-axis). Recent work indicates that the time spent in the ‘zone of (epigenetic) instability’ could determine the degree of rejuvenation of an old cell, as measured by methylation and transcription clocks (Gill et al., 2022); the optimum time spent in the zone (trajectory A) results in an age reprogrammed cell that is young (red), whereas a longer time spent in the zone (trajectory B) results in an age reprogrammed cell that is older (less young; given in purple). Based on a model by Manukyan and Singh (2012).

Several studies have shown that OSKM-driven age reprogramming can take place without overt de-differentiation and loss of cellular identity (Lu et al., 2020; Manukyan and Singh, 2014; Ocampo et al., 2016; Sarkar et al., 2020; reviewed by Simpson et al., 2021). Although this is indeed the aim of age reprogramming, these studies did not address whether temporary changes take place at the molecular level. Transient de-differentiation is proposed to be part of the age reprogramming process, with de-differentiation being necessary for cell types that are refractory (Manukyan and Singh, 2012; Singh et al., 2019). In this model, an aged cell transiently de-differentiates after introduction of OSKM reprogramming factors and passes through a ‘zone of (epigenetic) instability’, but once OSKM expression is stopped the cell returns to its original cellular identity having now undergone age reprogramming (Fig. 2, trajectory A). Two recent *in vitro* ‘-omic’ studies have explored the idea of transient de-differentiation during age reprogramming (Gill et al., 2022; Roux et al., 2022) and uncovered some surprising results that point to questions that might require future investigation.

In the first study, a lentiviral system was used to transiently express OSKM reprogramming factors for 3 days in aged adipogenic and mesenchymal stem cells (Roux et al., 2022). OSKM expression was then stopped and, 3 days later, single cell transcriptomic profiles were generated and subjected to pseudo-time analysis, which enabled the trajectory of partial reprogramming to be deduced using the relationships amongst the profiles. Partial reprogramming was shown to induce de-differentiation with suppression of cellular identity and activation of pluripotency genes (even of some late-stage pluripotency genes) in both adipogenic and mesenchymal stem cells. Cells then re-differentiated and re-acquired their original somatic identity along with a more youthful gene expression profile, with some of the features of youthful gene expression being maintained up to 10 days after OSKM reprogramming was stopped. This is consistent with the proposed model (Fig. 2, trajectory A). By contrast, a second *in vitro* study (Gill et al., 2022) gave results that diverged from the model but in an informative way. In this study, OSKM factors were expressed for 10, 13, 15 or 17 days in aged human dermal fibroblasts, whereupon these cells underwent morphological de-differentiation, forming colony-like structures typical of reprogramming intermediates. After culture for 4-5 weeks without OSKM, the reprogramming intermediates returned back to their original elongated fibroblast cellular identity. A transcriptomic analysis of the resultant transiently reprogrammed fibroblasts was then used to analyse rejuvenation of the cells, based on an ‘aging clock’ (see Box 1) that measures ‘transcription age’. This revealed a reduction in the mean transcription age of the cells of ∼20-30 years on days 10 to 13 of reprogramming. However, reprogramming for days 15 and 17 did not reduce transcription age further. A similar picture emerged from an epigenomic analysis, revealing a reduction in epigenetic age (eAge) of ∼30 years after 13 days of reprogramming, with days 15 and 17 eliciting a less pronounced reduction in eAge. These data indicate that there may be an optimum degree of de-differentiation, beyond which return to the original somatic identity does not rejuvenate cells further (Fig. 2; compare trajectory A with B). Based on this study, it was suggested that there are (undefined) cellular stresses when OSKM-mediated reprogramming is stopped after day 13 (on days 15 and 17) and this reduces the extent to which transiently reprogrammed fibroblasts are rejuvenated (Gill et al., 2022).

**Box. 1. Ageing clocks**

Ageing clocks attempt to disentangle biological age from chronological age (reviewed by Palmer, 2022). Estimation of biological age is important because it enables the identification of genetic and environmental factors that impact the ageing process, as well as interventions that can reverse or slow down the process itself. Clocks can be constructed from any biological system that varies with age, including age-related changes in:

- DNA methylation, from which methylation clocks can be constructed that determine epigenetic age (eAge).
- Gene expression, from which transcription clocks can be constructed that determine transcription age.
- Telomere length, from which telomere clocks can be constructed that determine telomere age.
- Secreted cell-surface glycome, which can be used to construct glycomic clocks, which can determine glycomic age.
- Extracellular vesicles such as exosomes, which can be used to construct exosomal clocks that can determine exosomal age.
- Biochemical factors in the blood/serum (biomarkers) that can be used to construct metabolomic clocks that can determine metabolomic age.

**Fig. 3.**
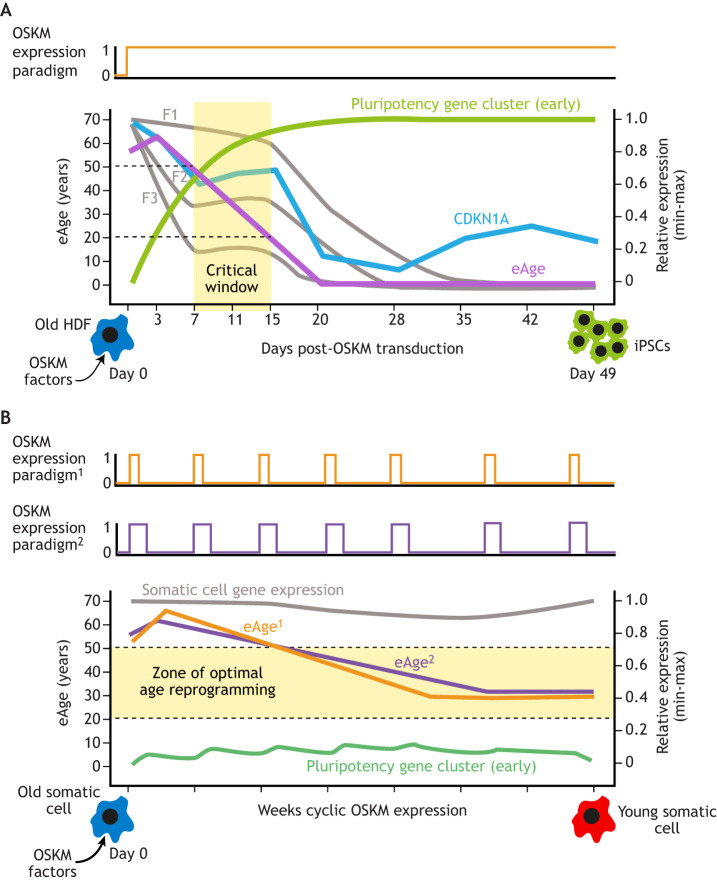
**Defining a ‘critical window’ for age reprogramming and designing reprogramming regimes.** (A) The classical virally-transduced OSKM expression paradigm is shown at the top. The ‘critical window’ (highlighted in yellow) extends from day 7 to 15. This window was chosen, *inter alia*, on the basis of a plateau of expression for three clusters of fibroblast-specific genes (F1, F2 and F3) in the face of eAge falling from 50 to 20 years; cellular identity (defined by expression of F1, F2 and F3) is stable while eAge falls. Also shown is expression of the (early) pluripotency gene cluster (green) and the cell-cycle inhibitor p21 (CDKN1A; blue) that initially falls but then exhibits a rising plateau of expression during the ‘critical window’. Accumulation of senescence-associated gene products towards the end of the ‘critical window’ may provide an explanation for why transient OSKM expression beyond day 13 results in reduced age reprogramming. Based on a model by Simpson et al. (2021) and Singh et al. (2019). HDF, human dermal fibroblast. (B) Cyclic OSKM expression paradigms can be used to develop cell-type specific age reprogramming regimes. Based on the ‘critical window’ in A, a ‘zone of optimal age reprogramming’ can be defined whereby maintaining eAge between 20 and 50 enables age reprogramming to proceed without suppression of somatic identity. The ‘plasticity’ of cellular phenotype determines the type of cyclic expression paradigm to be used. Expression paradigm 1 (eAge^1^; orange) has a cyclic regime in which OSKM is expressed for 1 day followed by no expression for 6 days. This would be appropriate for hepatocytes, for which expression for more than 1 day is known to be deleterious. Expression paradigm 2 (eAge^2^; purple), using 2 days of OSKM expression and 5 days of no expression, could be used for cardiomyocytes that are more refractory to OSKM reprogramming and require a longer ‘burst’ of OSKM expression. For both paradigms, the aim is to keep eAge between 20 and 50, where there is low level expression of the early pluripotency gene cluster (green) that follows the cyclic expression paradigm, while levels of somatic cell gene expression (grey) remain elevated. Once eAge is in the ‘zone of optimal age reprogramming’ it can be kept there if the ‘bursts’ of OSKM are less frequent, as shown. Based on a model by Singh et al. (2019).

An alternative explanation for the above finding comes from the identification of a ‘critical window’ in which age reprogramming proceeds without irreversible loss of somatic identity. This window, which was identified in an *in silico* analysis of a 49-day iPSC reprogramming time course, is predicted to occur provisionally between days 3 and 13 (Olova et al., 2019; Simpson et al., 2021). The critical window represents a period during the trajectory from somatic to iPSC fate in which age reprogramming, as measured by a decline in eAge, takes place with little change in somatic identity, as measured by fibroblast-specific gene expression (Fig. 3A; Singh et al., 2019). The study by (Gill et al., 2022) showed that the degree of transcriptomic and epigenetic rejuvenation is lower if reprogramming is stopped after day 13, on days 15 and 17. An explanation for this could come from the observation that a rising plateau of senescence-associated gene expression coincides with the critical window, between days 11 and 15 (Fig. 3A; Olova et al., 2019; Singh et al., 2019). Due to an accumulation of senescence-associated gene products, reprogramming beyond day 13 could return transiently reprogrammed fibroblasts to a more aged phenotype. If this is the case, judicious manipulation of senescence markers (e.g. delaying the ‘plateau’ of senescence-associated expression) might extend the critical window and allow a greater degree of age reprogramming.

Whether transient de-differentiation is a requirement for age reprogramming *in vivo* is an open question. We have yet to see a well characterised *in vivo* experiment in which partial reprogramming transiently de-differentiates an old cell and, after reprogramming is stopped, the cell returns to its original, now rejuvenated, cellular identity. We have come close in an experiment in which a 1 week ‘burst’ of OSKM expression in 55-week-old mice resulted in transient histological changes in the pancreas, intestine and stomach, but not in the liver and spleen (Chondronasiou et al., 2022). The changes were reversed in pancreata 2 weeks after OSKM expression was stopped and, importantly, the ‘burst’ of OSKM was sufficient to rejuvenate epigenetic, transcriptomic and metabolomic characteristics in several tissues, including pancreata, and in the serum (Chondronasiou et al., 2022). The effects of *in vivo* partial reprogramming on cardiomyocyte (CM) and liver regeneration after injury have also been analysed but, although these studies provided more molecular details of transient de-differentiated states, the use of young animals did not allow the degree of rejuvenation of the re-differentiated cells to be determined (Chen et al., 2021; Hishida et al., 2022). In order for the latter to be measured, the same studies should be repeated on aged animals.

## Is age reprogramming safe and, if so, how robust is it?

De-differentiation resulting in erasure of cellular identity in iPSCs followed by re-differentiation to generate specific cell types is accompanied by a number of well-known hazards that have limited the utility of the de-/re-differentiation cycle for regenerative therapies (Manukyan and Singh, 2012). Moreover, the *in vivo* induction of iPSCs by uninterrupted expression of OSKM has been shown to increase the incidence of teratomas (Abad et al., 2013). Age reprogramming was posited to avoid these difficulties because the aim is to retain cellular identity and simply rejuvenate the specialised differentiated function(s) of a cell. However, uncertainty remains as to whether age reprogramming, or indeed any application of partial reprogramming, is safe, especially in the context of aged tissues that have increased numbers of senescent cells. Senescent cells *in vivo* engage in paracrine signalling to non-senescent cells via the senescence-associated secretory phenotype, thereby augmenting OSKM-driven reprogramming and increasing the incidence of teratomas (Chiche et al., 2017). Further, OSKM expression can increase teratoma formation if extrinsic barriers to partial reprogramming are removed (Melendez et al., 2022). These and other potential hurdles highlight that we have some way to go before age reprogramming can become a routine approach to regenerative medicine.

There are few experiments to draw upon, but the best characterised approach to *in vivo* age reprogramming is the cyclic OSKM expression paradigm (Browder et al., 2022; Ocampo et al., 2016; Rodríguez-Matellán et al., 2020). An early *in vivo* study showed cyclical OSKM expression (2 days ‘on’, 5 days ‘off’) in a mouse LAKI premature ageing model can rejuvenate four hallmarks of ageing – epigenetic modifications, cellular senescence, DNA damage and mitochondrial dysfunction – with no gross histological changes (Ocampo et al., 2016). A similar 3 days ‘on’, 4 days ‘off’ regime in 6-month-old wild-type mice, continuing for 4 months, has also been shown to rejuvenate age-related characteristics in dentate gyrus cells, without increasing mortality (Rodríguez-Matellán et al., 2020). Most recently, cyclic OSKM expression (2 days ‘on’, 5 days ‘off’) in physiologically-aged 15-month-old mice for 7 months was shown to rejuvenate skin and reduce eAge without affecting overall health, as evidenced by normal blood counts, no loss of performance in neurological tests and no gross histological changes (Browder et al., 2022). Maintaining low levels of cyclic OSKM expression also appears to be important: cyclical OSKM expression for 35 cycles (35 weeks) in mice harbouring a single copy of the OSKM transgene does not increase the incidence of teratomas, yet eight cycles of OSKM expression in animals possessing two copies of the transgene increases cell proliferation and teratoma formation in the liver, kidney and pancreas (Ocampo et al., 2016).

The future development of age reprogramming therapies based on cyclic OSKM expression will have to take into account the observation that tissues are not equally reprogrammable. For example, adult hepatocytes are more ‘plastic’ compared with CMs. Hepatocytes exhibit spontaneous cellular reprogramming during liver regeneration, and hepatocyte-specific OSKM expression for just 2 days is lethal (Hishida et al., 2022). By contrast, CMs require 6 days of CM-specific OSKM expression before exhibiting signs of de-differentiation, and 12 days before animals become increasingly sick and die (Chen et al., 2021). One approach that could be tailored to accommodate the susceptibility of cell types to OSKM reprogramming *in vivo* is to use cyclic OSKM expression to maintain eAge within a ‘zone of optimal age reprogramming’ (Fig. 3B; Singh et al., 2019). Within this zone, age reprogramming proceeds while avoiding the hazards of de-differentiation (e.g. dysplasia and teratomas; Abad et al., 2013). Cell-type specific cyclic regimes could thus be designed such that a differentiated cell type having greater cellular ‘plasticity’ (e.g. liver cells; Hishida et al., 2022) is exposed to a shorter ‘burst’ of OSKM expression compared with other cell types that that are more refractory (e.g. CMs; Chen et al., 2021) and require a longer ‘burst’ of OSKM expression (Fig. 3B). In addition, the safety of the ‘bursts’ could be augmented through use of RNA electroporation that ensures transient expression of OSKM (NANOG, LIN28; NL) (Sarkar et al., 2020) or by use of newly discovered chemical compounds that are able to replace OSKM (Guan et al., 2022). The recent work of Guan et al. (2022) is particularly exciting, as it sets the stage for the development of lipophilic compounds that can cross cell membranes and regulate reprogramming factors. Topical application of such compounds to aged tissues (e.g. skin) could enable *in vivo* age reprogramming in humans, although care would have to be taken to avoid irreversible de-differentiation with concomitant neoplastic risk.

In a systematic search for safer age reprogramming regimes, a screen of different OSKM reprogramming combinations showed that the c-MYC oncogene-free OS combination restored a youthful transcriptional profile in mesenchymal cells with a reduced effect on the loss of cellular identity, indicating that this combination may minimise the risks of suppressed cellular identity and potential neoplastic transformation (Roux et al., 2022). Expression of the larger OSK set, again lacking c-MYC, in the eyes of middle-aged 11-month-old mice restored visual acuity that was associated with a more youthful gene expression profile; there was also no increase in tumour incidence (Lu et al., 2020). Transient expression of the non-canonical reprogramming factor NANOG has also been shown to rejuvenate senescent myoblasts without increased tumour incidence (Shahini et al., 2021).

The other side of the coin is whether age reprogramming is robust. That is, do age reprogrammed cells re-age at the rate of young cells or do they age more rapidly? And is the rejuvenated state stable? Early work investigating these issues was not encouraging. Epigenetic rejuvenation of senescent cells lasted but a few days (Manukyan and Singh, 2014). In addition, cycling of OSKM (Ocampo et al., 2016) was required to ameliorate ageing phenotypes in LAKI progeroid mice, and similar cycles of OSKM expression were necessary to maintain rejuvenation of several senescence-associated characteristics in nucleus pulposus cells of the vertebral disc (Cheng et al., 2022). However, a more recent *in vivo* experiment addressed the robustness of age reprogramming using a longer ‘burst’ of OSKM expression in a premature ageing (progeria) mouse model. In this approach, a single 2.5 week ‘burst’ of Dox-induced OSKM expression in 2-month-old mice heterozygous for both an OSKM transgene and the LAKI (*Lmna^G606/+^*) mutation was shown to extend lifespan by 15%; lifespan extension was also observed in non-progeria mice (Alle et al., 2022). Remarkably, 6 months after OSKM expression was stopped in progeria mice, lean mass was retained, motor skills were enhanced and the integrity of the bones, lungs, spleen, kidney and skin was preserved compared with untreated controls (Alle et al., 2022). A DNA methylation signature that correlated with rejuvenation was also propagated in rejuvenated tissues 6 months after the 2.5 week ‘burst’ of OSKM expression. A more detailed study of age reprogramming in physiologically-aged mice could be undertaken using OSKM reprogrammable mice that have been engineered to exclude OSKM expression from the liver and intestine, where expression results in early mortality (Parras et al., 2022 preprint). These mice should enable longer term induction of OSKM and could be used to test the effect of an extended period of OSKM expression early in life on health benefits later in life.

## Are there factors other than OSKM that could be used for age reprogramming?

During development, there is a gradual restriction in developmental potential, whereby the descendants of pluripotent epiblast cells include oligo-potential cells that in turn give rise to mono-potential terminally-differentiated cells that populate adult tissues (Keller, 2005). OSKM factors reverse these restrictions and drive differentiated cells towards pluripotency; lineage-specific gene expression is silenced with loss of differentiated cellular identity, and the resultant iPSCs exhibit expanded developmental potential. Interestingly, factors that reverse the later restriction, driving mono-potential cells towards oligo-potency, can also be used for age reprogramming. For example, expression of *Msx1* in mono-potential myocytes silences myocyte-specific gene expression leading to de-differentiation and expansion of developmental potential (Odelberg et al., 2000). *Msx1* can also restore youthful gene expression when transiently expressed in aged myogenic cells (Roux et al., 2022). It would appear that genes possessing the ability to suppress differentiated cellular identity (e.g. by silencing lineage-specific gene activity) and expand developmental potential are likely to regulate age reprogramming. In this regard, it will be of interest to elucidate the pathway by which cyclic expression of the FOXM1 transcription factor delays natural ageing and extends lifespan (Ribeiro et al., 2022).

Good candidates for the regulation of age reprogramming are the highly-conserved Polycomb Group (PcG) proteins (Grossniklaus and Paro, 2014). PcG proteins are known to regulate passage through the maturation phase of iPSC reprogramming by silencing lineage-specific gene expression and subsequently maintaining the pluripotent ground state (Khazaie et al., 2016). In mammals, PcG proteins are found in two complexes: PRC1 and PRC2. The PRC1 complex contains the PcG eponym, Pc (aka CBX proteins), which binds the epigenetic modification H3K27me3, the Bmi1/Mel18 proteins and the Ring1A/B E3 ubiquitin ligase. The canonical PRC2 complex contains the H3K27 histone methyl-transferase Ezh2/1, Eed and Suz12 proteins. Both complexes silence gene activity, via mono-ubiquitylation of lysine 119 of histone H2A by PRC1, and via mono-, di- and tri-methylation of H3K27 by PRC2. PcG domains that are assembled from H3K27me3-marked chromatin and PRC1/2 complexes are termed facultative heterochromatin (Miller et al., 2017).

PcG proteins can substitute for classical OSKM reprogramming factors. For example, the combination of Bmi1 and Oct4 can reprogramme fibroblasts into iPSCs (Moon et al., 2011) and the efficiency of iPSC reprogramming is enhanced by Ezh2 expression (Ding et al., 2014). There are thousands of PcG target loci in the genome, many of which overlap with CpG islands (CGIs) that are generally hypomethylated (Ku et al., 2008). DNA hypomethylation is mediated by Tet dioxygenases that interact with PRC2 and both mutually recruit each other to maintain hypomethylation at CGIs (Li et al., 2018). Notably, Tet1 can substitute for Oct4 in the classical OSKM reprogramming set (Gao et al., 2013), and knockdown of *Tet1* or *Tet2* stops age reprogramming (Lu et al., 2020). Important questions for the future will be whether PRC1/2-mediated facultative heterochromatinisation regulates age reprogramming and, given that PRC2 can recruit Tet1/2 proteins to CGIs, whether facultative heterochromatinisation can affect eAge. These questions could be answered by manipulating topoisomerase IIa (TopoIIa) activity. TopoIIa is necessary for OSKM-driven partial reprogramming (Hishida et al., 2022), is known to regulate assembly of H3K27me3-marked facultative heterochromatin (Miller et al., 2017) and, in embryonic stem cells (ESCs), localises to CGI promoters that possess H3K27me3 as part of H3K27me3/ H3K4me3 bivalent marks (Thakurela et al., 2013).

## Does age reprogramming occur during vertebrate development?

Tracking eAge during pre-/early post-implantation in the mouse showed that the eAge of the newly-fertilised zygote gradually decreases to reach its lowest levels at around embryonic day (E) 6.5 to E7.5 as embryos undergo gastrulation (Gladyshev, 2021; Kerepesi et al., 2021). This remarkable observation indicates that the zygote is actually ‘old’ and becomes ‘younger’: the embryo is rejuvenated during pre-/early post-implantation development, as measured by eAge, reaching its most youthful state around gastrulation. During gastrulation, the epiblast undergoes dramatic morphogenetic movements to form the three germ layers – ectoderm, mesoderm and endoderm (Wolpert and Skinner, 1993) – that contain progenitors for the future body plan that is laid down during a highly conserved stage of development called the ‘phylotypic stage’. This represents a stage in development when an animal most closely resembles other species. In vertebrates, the identification of a precise phylotypic stage that is identical in all species has been elusive. Instead, there is thought to be a phylotypic ‘period’ or ‘progression’ (Richardson, 1995) that roughly corresponds to the period of organogenesis, when numerous organ primordia are developing from the three germ layers, followed by overt organ/tissue-specific differentiation.

We propose that age reprogramming takes place during pre-/early post-implantation development, leading up to phylotypic progression, and is necessary to ensure that the embryo is rejuvenated and optimally primed to direct development. There is *in vivo* evidence derived directly from embryos as well as indirect evidence from *in vitro* studies on iPSCs/ESCs to indicate that the hallmarks of ageing, including telomere length, mitochondrial function, DNA repair and epigenetic drift (López-Otín et al., 2013), are rejuvenated during pre-/early post-implantation development. Telomeres in the oocyte are short and are extended during the early cleavage divisions by a telomerase-independent mechanism involving sister-chromatin exchange (SCE; Liu et al., 2007). It is only around the blastocyst stage that telomerase activity maintains telomere length established by SCE. It is also known that numbers of mitochondria increase around implantation (May-Panloup et al., 2016), and that mitochondrial numbers and bioenergetic activity are subject to age-related decline in old oocytes (May-Panloup et al., 2016). Moreover, work on iPSCs has shown that passage through the embryonic iPSC stage can rejuvenate the bio-energetic functions of old mitochondria (Lapasset et al., 2011). Partial reprogramming has also shown that rejuvenation of mitochondrial function is separable from de-differentiation (Ocampo et al., 2016; Sarkar et al., 2020). Finally, there is *in vitro* evidence showing that ESCs can repair DNA damage more efficiently than somatic cells (Fu et al., 2017), that ‘bursts’ of two-cell (2C)-like expression in ESCs help to maintain telomere length and genome stability (Ko, 2016), and that partial reprogramming also rejuvenates DNA repair activity (Ocampo et al., 2016). Regarding the hallmark of epigenetic drift, epigenetic rejuvenation reverses age-related changes in the epigenome and there is good *in vitro* evidence that epigenetic rejuvenation is a key driver of age reprogramming. Initial studies showed that the age-related decline of the H3K9me3 histone modification could be restored by partial reprogramming (Cheng et al., 2022; Gill et al., 2022; Ocampo et al., 2016; Rodríguez-Matellán et al., 2020; Sarkar et al., 2020). More importantly, if restoration of H3K9me3 levels is inhibited, OSKM-driven age reprogramming is stopped (Ocampo et al., 2016). These findings highlight the importance of epigenetic rejuvenation in the process of age reprogramming, which we discuss in detail below.

## What epigenetic changes occur during age reprogramming in the early embryo?

**Fig. 4.**
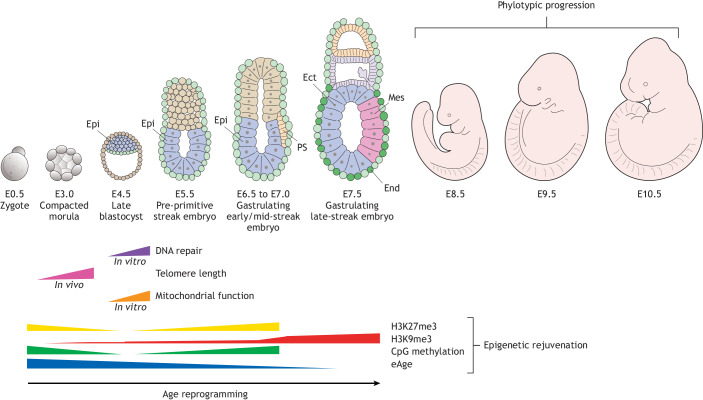
**Age reprogramming during pre-/early-post implantation development in the mouse.** The hallmarks of ageing, telomere attrition, mitochondrial dysfunction, DNA repair and epigenetic drift are rejuvenated during early development: telomeres are extended *in vivo* during pre-implantation development; there is *in vitro* evidence that mitochondrial function is rejuvenated in differentiated iPSC derivates and via partial reprogramming; DNA repair is elevated in ESCs; and there is epigenetic rejuvenation of histone and DNA modifications during pre-/early-post implantation development that results in a global repressive epigenetic landscape in or around gastrulation, before entry into the phylotypic progression (note that the embryos depicted at E8.5 and older give a rough idea of when phylotypic progression occurs, although an exact phylotypic ‘stage’ for vertebrates has been difficult to identify). H3K9me3 increases and becomes enriched over promoters, gene bodies and termination sites in mesodermal and endodermal cells at E8.25 (Nicetto et al., 2019; Wang et al., 2018). Global H3K27me3 histone modification and CpG methylation levels reach maximal levels at around E6.5 (Auclair et al., 2014; Zheng et al., 2016), although many PRC2-targeted CGIs are demethylated through the PRC2-mediated recruitment of Tet dioxygenases (Li et al., 2018; Zhang et al., 2018). Remarkably, resetting of H3K9me3, H3K27me3 and CpG methylation broadly coincides with the eAge ground state at around E6.5-E7.5. Ect, ectoderm; End, endoderm; Epi, epiblast; Mes, mesoderm; PS, primitive streak.

**Fig. 5.**
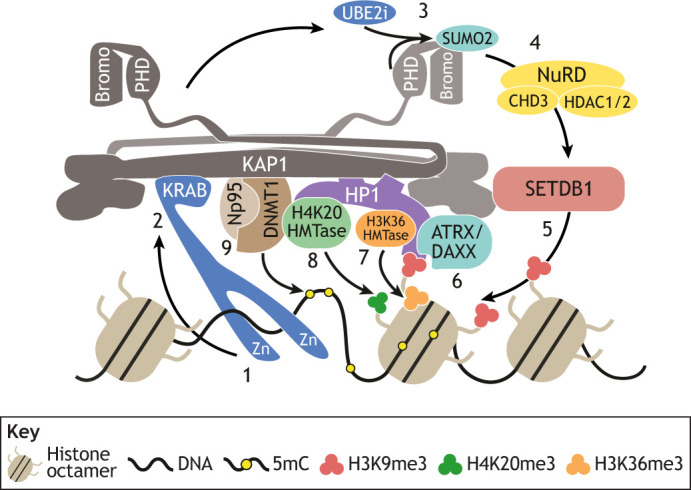
**Heterochromatin*-*like complexes safeguard cellular identity.** Small heterochromatin*-*like complexes can ‘nucleate’ the assembly of larger H3K9me3-marked heterochromatin*-*like domains. Targeting and formation of the complexes occurs in several steps. (1) KRAB-ZNF binds to its DNA binding site through its zinc-fingers (Zn). (2) The KRAB domain of KRAB-ZNF interacts with KAP1. One molecule of KAP1 binds to an HP1 dimer, which in turn binds to H3K9me3 (red circles). (3) The PHD domain of KAP1 is an E3 ligase that cooperates with UBE2i to sumoylate (SUMO2) the KAP1 bromodomain. (4) The sumoylated bromodomain is bound by the NuRD complex that deacetylates acetylated histones in preparation for histone methylation. (5) SETDB1 H3K9 HMTase interacts with the sumoylated (SUMO2) bromodomain and generates H3K9me3 (red circles). (6) The ATRX/DAXX complex is bound to KAP1, HP1 and H3K9me3. ATRX/DAXX incorporates replacement histone H3.3 into chromatin, thereby reinforcing nucleation. (7) HP1 recruits an H3K36me3 HMTase and generates H3K36me3 (orange circles). (8) HP1 recruits an H4K20 HMTase that generates H4K20me3 (green circles). (9) KAP1 binds to the maintenance DNA methylase DNMT1 and its co-factor Np95. DNMT1 maintains cytosine methylation at the site of assembly. Modified from Singh and Newman (2020). The interaction of HP1 with H3K36me3 HMTase has been described (Zaidan and Sridharan, 2020) and it is known that the heterochromatin*-*like complex can generate the H3K36me3 modification (Sripathy et al., 2006). Several components of heterochromatin*-*like complexes have been identified in screens as being important in safeguarding cellular identity (see Table 1).

Epigenetic rejuvenation during embryogenesis is characterised by the establishment of a global repressive epigenetic landscape that is fully elaborated in or around gastrulation. It is the establishment of this repressive environment that most likely results in the ‘ground-state’ of eAge that is achieved at this stage of early post-implantation development (Gladyshev, 2021; Kerepesi et al., 2021). There are three repressive epigenetic modifications, H3K9me3, H3K27me3 and CpG methylation, that have been studied in some detail (Fig. 4). For example, it has been shown that the levels and distribution of H3K9me3 are reset during mouse development (Nicetto et al., 2019; Wang et al., 2018). H3K9me3, along with the non-histone protein HP1, is a hallmark of constitutive heterochromatin, although both H3K9me3 and HP1 co-localise in euchromatic arms where they assemble large (up to several Mb) H3K9me3-marked heterochromatin-like domains (reviewed by Singh et al., 2020). H3K9me3 becomes enriched over promoters, gene bodies and termination sites, with the greatest number of genes marked by H3K9me3 present in non-committed mesodermal and endodermal cells on E8.25 (Nicetto et al., 2019; Wang et al., 2018). As development proceeds, H3K9me3-marked heterochromatin undergoes dramatic reorganisation (Nicetto et al., 2019), and H3K9me3-marked tissue/lineage-specific genes lose H3K9me3 upon differentiation into their respective lineages. Conversely, genes active in uncommitted mesodermal and endodermal lineages are silenced in differentiated cells and gain H3K9me3 (Nicetto et al., 2019). The silencing of lineage-inappropriate genes by H3K9me3-marked heterochromatin plays a crucial role in safeguarding cellular identity. Evidence for this comes from screens for genes that, when inhibited, increase OSKM-mediated reprogramming efficiency. Several of the genes identified encode components of small heterochromatin*-*like complexes that can ‘nucleate’ the assembly of larger H3K9me3-marked heterochromatin*-*like domains (Fig. 5; Table 1). These include KAP1, SETDB1 H3K9HMTase, SUMO-conjugating enzyme UBE2i, SUMO2, ATRX and DAXX proteins (Borkent et al., 2016; Cheloufi et al., 2015; Miles et al., 2017). Such heterochromatin*-*like complexes are targeted to specific sites by sequence-specific KRAB-zinc-finger proteins (KRAB-ZFPs), which represent the largest family of transcriptional repressors in mammals (Imbeault et al., 2017). The OSKM reprogramming screens also identified CAF1-p150 and -p160, proteins that are involved in the replication of H3K9me3-marked heterochromatin (Cheloufi et al., 2015). The importance of H3K9me3 in safeguarding cellular identity is underscored by the observation that reducing H3K9me3 levels through ectopic expression of an H3K9me3-specific demethylase in oocytes, or by knocking down the H3K9 methyltransferases Suv39h1/2, improves reprogramming efficiency of somatic cell nuclear transfer (Matoba et al., 2014; Table 1). Understanding precisely how H3K9me3-marked heterochromatin*-*like domains/complexes play a role in safeguarding cellular identity will need to be a focus for age reprogramming research, so that we can learn how to safely rejuvenate differentiated cells while maintaining cellular identity.

**Table 1.**
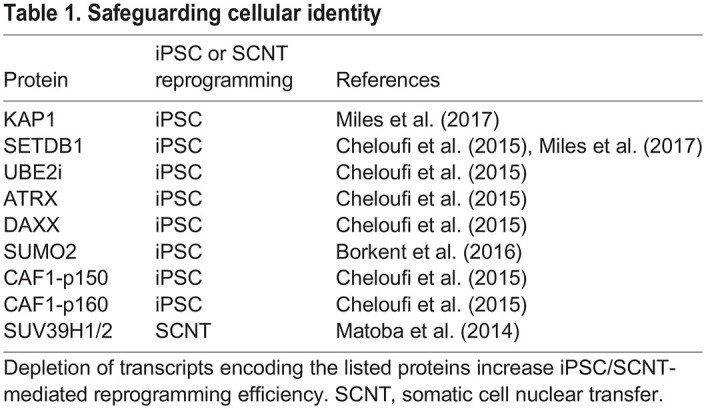
Safeguarding cellular identity

H3K27me3 and CpG DNA methylation show similar profiles of expression during development (Fig. 4) and there appears to be a close relationship between PcG proteins (particularly the PRC2 complex) and the Tet-mediated DNA demethylation system. As for H3K27me3, its levels at CGIs/promoters and more distal regions are highly dynamic during mouse embryogenesis (Zheng et al., 2016). There is a rapid increase of H3K27me3 levels at promoters of developmental genes soon after implantation (around E5.5) that is well established by E6.5 in the epiblast. As embryonic development proceeds, H3K27me3, like H3K9me3, undergoes a re-organisation such that it becomes lost from developmental genes such as the Hox genes that are required for the patterning of the anterior-posterior axis (Deschamps and Duboule, 2017). In differentiated cells, genes targeted by PRC2 complexes are resistant to age reprogramming, consistent with a role for H3K27me3 in silencing lineage-inappropriate genes and safeguarding cellular identity (Roux et al., 2022).

As mentioned above, PRC2 complexes interact with Tet dioxygenases and both mutually recruit each other to maintain hypomethylation at CGIs/promoters (Li et al., 2018). This mutual dependency at CGIs/promoters is intriguing, especially in the context of a recent survey of DNA methylation profiles from 185 mammalian species showing CpGs that change consistently with age predominantly reside in PRC2-binding sites and H3K27me3/H3K4me3 bivalent promoters (Lu et al., 2022 preprint). The gain of methylation at these sites as cells age could be explained by gradual loss of PRC2 targeting to CGIs/promoters with concomitant loss of Tet dioxygenases. Notably, knockdown of *Tet1* or *Tet2* stops OSK-driven age reprogramming (Lu et al., 2020), indicating that Tet-mediated DNA demethylation of methylated CpGs, presumably targeted by PRC2, is required for age reprogramming.

## Perspectives

As we have outlined here, recent studies have provided us with the first molecular insights into the concept of age reprogramming. We have highlighted how this process could potentially be leveraged in the context of regenerative medicine. Lipophilic compounds that regulate OSKM may be used in conjunction with knowledge of the ‘zone of optimal age reprogramming’ (Fig. 3B) to develop *in vivo* tissue-specific age reprogramming regimes in humans.

Epigenetic rejuvenation is a major driver of age reprogramming and reverses the age-related erosion of the epigenetic landscape (e.g. erosion of the levels and distribution of histone and DNA modifications). For a particular cell-type, epigenetic rejuvenation aims to return the epigenetic landscape back to the state present at the time the cell type first arose during development; the cell is rejuvenated while retaining its specialised, differentiated, functions. Being able to safely and robustly return the epigenotype of an aged cell to that found at its inception could have long lasting-effects and might explain how the rejuvenating effects of a single short ‘burst’ of OSKM early in life can still be detected 6 months later (Alle et al., 2022). In this context, it is of interest that the ‘immortal’ jellyfish, *Turritopsis dohrnii*, appears to achieve biological immortality through cycles of epigenetic rejuvenation involving the PRC2 complex that silences developmental genes (Pascual-Torner et al., 2022), similar to the way in which epigenetic rejuvenation establishes a global repressive nuclear environment during age reprogramming of the mammalian embryo (Fig. 4). It remains to be seen whether cycles of epigenetic rejuvenation by age reprogramming could confer biological immortality in mammals, including humans.

## Supplementary Material

10.1242/develop.200755_sup1 Supplementary information
