# Supplementary material for: Age reprogramming: cell rejuvenation by partial reprogramming
Source: Development. 2022 Nov 16;149(22):dev200755. doi: 10.1242/dev.200755 (PMC9845736; doi:10.1242/dev.200755)
Supplement: Supplementary information [file develop-149-200755-s1.pdf]

Table S1. Age reprogramming using partial reprogramming with OSKM(NL) reprogramming factors. Not shown are experiments where partial reprogramming has been used to enhance tissue regeneration after injury (*e.g.*, Chen et al., 2021; Wang et al., 2021; Hishida et al., 2022) and to promote trans-differentiation of one differentiated cell type to another (*e.g.* Maza et al., 2015).

Reference	OSKM(LN) expression paradigm	Physiological/cytological characteristics	Molecular characteristics
(Manukyan and Singh, 2014)	Expression of OSKM+LIN28 from “piggy-bac” vector in senescent human diploid fibroblasts under conditions that are non-permissive for iPS cell induction.	Loss of β-galactosidase senescence marker on day 9 after introduction of OSKML.	Mobility of HP1β rejuvenated, without de-differentiation, on day 9 after introduction of OSKML
(Ocampo et al., 2016)	OSKM was expressed from a single *dox*-inducible poly-cistronic cassette (i4FA transgene). OSKM was expressed *in vivo* and in *in vitro*.<br><br>*In vivo* expression followed a cyclical regime - 2 days “on” and 5 days “off”. This was done in WT 4F mice and in LAKI prematurely-aging (LAKI 4F) mice to investigate the effects in ameliorating age-related phenotypes. The regenerative capacity in physiologically-aged mice was also assessed using the same cyclical expression regime.<br><br>The effects of short-term *dox*-induced expression (2 or 4 days) of OSKM in LAKI 4F tail tip fibroblasts (TTFs) was also investigated.	Cyclic expression of OSKM in WT mice heterozygous for the 4F transgene for 35 cycles (weeks) did not result in weight loss or increased mortality. There were no signs of dysplasia or cancer development.<br><br>Cyclic expression of OSKM in LAKI 4F mice beginning at 8 weeks of age resulted in an increase in median and maximal lifespan of LAKI mice. No teratomas were found.<br><br>LAKI 4F mice showed significant improvement in age-related histological changes. These included: a reduction of spinal curvature, gross improvement of the gastrointestinal tract, improvement of the gross age-related cytology of spleen, kidney and stomach. Senescence-associated β-galactosidase expression was also reduced in liver. Also rescued were the macroscopic involution of the spleen, depletion of the lymphoid white pulp in germinal centres. The skin also showed increased epidermal and dermal thickness and decreased keratinization. Age-related cardiovascular changes were also reversed.	Cyclic expression of OSKM in LAKI 4F mice resulted in down regulation of stress response genes in the p53 pathway and age-related *interleukin-1a* in liver, stomach and kidneys. The levels of the histone modifications H3K9me3 and H4K20me3 were restored in the kidney and spleen.<br><br>LAKI 4F TTFs showed reduced γ-H2AX foci and p52 biding – both are markers of DNA damage response. LAKI 4F TTFs showed improved mitochondrial function as measured by reduced production of mitochondrial reactive oxygen species. The p53 stress pathway, including $p16^{INK4a}$, $p21^{CIP1}$, *Atf3*, *Gadd45B* and senescence-associated metalloprotease *MMP13* and *interleukin-6* all showed downregulation.<br><br>LAKI 4F TTFs also showed restoration of H3K9me3 and K20H4me3. Importantly it was shown that incubation of LAKI 4F TTFs with chaetocin (an inhibitor if H3K9 methyltransferases) prevented restoration of H3K9me3 levels, reduction of DNA damage and nuclear envelope defects that are induced by OSKM expression. This result indicates that changes in the epigenome is a driver of ageing and its rejuvenation is a key component in age reprogramming.
(Sheng et al., 2018)	SOX2 and c-MYC (SM) were introduced into peripheral blood lymphocytes (PBCs) by infection using a non-integrative temperature-sensitive Sendai virus (SeV) vector. After expression of SM in PBCs for up to 2 weeks elimination of the SeV vector was achieved by culturing cells at 39°C for 3-4 weeks.	Upon SeV infection with SM, infected cells were cultured in medium that promotes induced neural stem cell (PB-iNSC) conversion.<br><br>PB-iNSC possess the characteristic expanded tri-potential phenotype of bone fide NSCs with the ability to generate neurones, astrocytes and	These experiments indicate that the eAge reprogramming requires some degree of de-differentiation as evidenced by the association of reduced eAge with expanded developmental potential of PB-iNSCs as compared to iNs.<br><br>There was no control for the effect of the lengthy

		oligodendrocytes. eAge in low-passage iNSCs was reduced to 5.5% and 39.4% of the parental PBCs.<br><br>In contrast to PB-iNSCs, induced neurones (iNs) generated by forced expression of neurogenic transcription factors in mouse and human fibroblasts are mono-potential and retain age-associated transcriptomic and epigenetic signatures.	"heat-shock" used to eliminate the SeV vectors on eAge.
(Olova et al., 2019)	*in silico* analysis of a previously published 49-day OSKM iPS reprogramming time course on human dermal fibroblasts (HDFs). The time course enabled the dynamics of changes in eAge, fibroblast-specific gene expression, pluripotency gene expression, expression of senescence-associated markers and developmental genes to be investigated.	This was an *in silico* analysis.	Starting on day 3 after introduction of OSKM there is a steady decrease in eAge of 3.8 years per day, reaching zero by day 20. Zero is reached on the same day that both early (cluster 1) pluripotency markers and several key developmental genes reach maximal expression levels.<br><br>Fibroblast-specific genes (clusters 1, 2 and 3) showed an immediate decline in expression that subsequently plateaued from day 7 until day 15. Notably, expression of several key senescence markers peaked between days 11 and 15.<br><br>These results indicate that eAge reprogramming and developmental reprogramming are separable and there is a safe window where rejuvenation can be achieved with a minimized risk of cancer.<br><br>It was noted that the plateauing of fibroblast-specific expression at the time that expression of senescence markers peaks might indicate that the latter could delay the loss of somatic identity.
(Sarkar et al., 2020)	A cocktail of OSKM+LIN28+NANOG (OSKMLN) mRNAs were transfected for 4 consecutive days into aged human dermal fibroblasts (60-90 years of age) and endothelial cells (50-65 years of age) resulting in transient expression of reprogramming factors.<br><br>The same cocktail of mRNAs were transfected into chondrocytes from six elderly (60-70 years of age) patients for 2 to 3 days.	There was an increase in mitochondrial membrane potential and a reduction in mitochondrial reactive oxygen species (ROS) in aged OSKMLN-treated fibroblasts. A less significant increase in mitochondrial membrane potential was observed in OSKMLN-treated endothelial cells along with no change in mitochondrial ROS. These data indicate there may be cell-type specific rejuvenation of mitochondrial function.	In aged OSKMLN-treated fibroblasts and endothelial cells the levels of the epigenetic modification H3K9me3 is rejuvenated along with HP1γ and nuclear lamina support protein, LAP2α.<br><br>An RNA-Seq analysis showed that transient expression of OSKMLN in aged fibroblasts and endothelial cells resulted in more youthful transcriptomic profiles.

Development • Supplementary information

	Similarly, aged murine muscle stem cells (MuSCs; 20-24 months) and human MuSCs (ages ranging 10-80years) were transfected with the same six mRNAs for 2 days.	In aged OSKMLN-treated fibroblasts and endothelial cells autophagosome rejuvenation was observed. However, OSKMLN treatment showed significant rejuvenation of proteasomal activity in endothelial cells but not in fibroblasts. Similarly, senescence-associated β-galactosidase activity decreased in endothelial cells but not in fibroblasts.<br><br>OSKMLN-treated aged chrondrocytes showed increased proliferation, enhanced mitochondrial function, reduction in levels of inflammatory factors and no loss of cellular identity.<br><br>OSKMLN-treated murine MuSCs *in vitro* have greater myogenic potential without affecting myogenic fate. *In vivo*, transiently reprogrammed aged murine MuSCs have improved tissue regenerative potential and can restore physiological function of aged muscles to that of youthful muscles.<br><br>OSKMLN-treatment of aged human MuSCs restores their potency *in vivo* to a degree similar to that of young MuSCs, without compromising their fate.	Using the pan-tissue Horvath methylation clock the eAge was reduced by 4.94 years in endothelial cells. A less pronounced eAge rejuvenation (1.84 years) was observed in fibroblasts.
(Rodríguez-Matellán et al., 2020)	OSKM was expressed from a single *dox*-inducible poly-cistronic cassette (4F transgene). There were two treatment regimens that were used to test the effect of transient OSKM expression on age-dependent changes in dentate gyrus cells. First, expression was continuous for 12 days in 6-month old mice (this protocol resulted in increased mortality). Second, weekly cycles of 3 days OSKM expression "on" followed by 4 days expression "off" starting at 6-months old until 10 months of age (there was no mortality using this second paradigm).<br><br>The effect of OSKM expression on adult hippocampal neurogenesis (AHN) was investigated. AHN takes place between the dentate gyrus and the hilus in the sub-granular zone and declines with age.	Using the first treatment regime an increase in the migration of cells positive for the late AHN marker doublecortin (DCX). Migrating cells also expressed higher levels of calretinin that could reverse the increase in calcium levels that have been observed in aged neurones.<br><br>Using the second regime, there was increased migration of DCX+ve cells, as observed with the first treatment regime. There was no increase in number of DCX+ve cells. In the molecular layer of the dentate gyrus the age-related decline in GluN2B was restored in mature neurones. The GluN2B subunit is a constituent of the NMDA receptor and its increase greater synaptic plasticity and could explain the partial improvement in object recognition in OSKM-expressing mice.	Using the first treatment regimen, the age-related decline in H3K9me3 was increased in migrating calretinin positive cells.<br><br>Using the second treatment regimen the age-related decline in H3K9me3 was restored in mature GluN2B-positive neurones.

Development • Supplementary information

(Lu et al., 2020)	Oct4, Sox2 and Klf4 (OSK) were expressed in retinal ganglion cells (RGCs) by intravitreal injection of *dox*-regulatable AAV2-OSK vectors.<br><br>Using this AAV2-based delivery system a few age-independent experiments were undertaken. These experiments showed:<br>(i) OSK expression did not induce tumours or structural changes (indicative of loss of cellular identity) in the retina, even if OSK expression was continuous for 15 months.<br>(ii) OSK expression can stimulate axon regeneration in a nerve crush model.<br>(iii) Knock-down of either Tet1 or Tet2 blocked OSK-dependent RGC survival and axon regeneration.<br>(iv) Over expression of TET1 catalytic domain had no protective or regenerative effect indicating that global DNA demethylation is necessary but not sufficient to protect and regenerate axons.<br>(v) OSK expression in human neurones counter acted axonal loss and eAge acceleration. OSK-mediated recovery was Tet2 dependent as it is in the mouse system.<br>(vi) OSK expression reverses axon density and vision-loss in a glaucomatous injury model.<br><br>Two age-related experiments that investigated the effects of OSK expression on rejuvenation were undertaken:<br><br>1. The effect on eAge of nerve crush and OSM expression 4 days post injury was investigated. eAge was assessed using a ribosomal DNA methylation clock.<br>2. OSM was expressed (tet-on) for 4 weeks in mice either 3-months (young mice) or 11-months (old mice) of age.	Regarding the two age-related experiments:<br><br>1. 4 days after nerve crush there was an eAge acceleration measured by the ribosomal clock, which was counteracted by OSK expression; OSK expression also promoted RGC survival and axon regeneration with no sign of proliferation. Global changes in DNA methylation that result from nerve crush injury were also reversed by OSK expression; many of the genes affected are associated with light detection and synaptic transmission.<br><br>2. 4 weeks of OSK expression restored vision in naturally-aged 11 month old mice without an increase in axon density or RGC proliferation.<br><br>A caveat in these experiments was that no markers for de-differentiation were used to confirm that the effect observed (e.g. RGC survival and axon regeneration) were de-coupled from de-differentiation.	Regarding the second age-related experiment:<br><br>Restoration of vision in the (4 week) OSK-treated 11 month old mice was likely due to changes in the transcriptome: RNA-Seq analysis revealed that of the 464 transcripts that showed age-related changes in expression around 90% returned to youthful levels after OSK expression.<br><br>A DNA methylation “ageing” signature based on 1226 CpG was constructed and showed that OSK reversed the methylation signature in context of both ageing and injury. Notably, the signature CpGs were associated with genes involved in synaptic and neuronal processes and were enriched for binding of the Polycomb repressive complex 2 (PCR2) and its corresponding histone modification H3K27me3. It was noted that both PCR2 and H3K27me3 are known to recruit the TET enzymes.
(Shahini et al., 2021)	For *in vitro* experiments, NANOG was expressed in young (passage 2) and senescent (S cells; passage 12) human myoblasts using a lentiviral vector. NANOG was expressed in senescent cells for 5 (SN5), 10 (SN10) or 15 (SN15) days.	*in vitro* experiments showed that expression of NANOG in S5, S10 and S15 cells resulted in increased cellular proliferation and loss of the senescence-associated β-galactosidase expression compared to S cells. NANOG expression in S	*In vitro* experiments showed that expression of NANOG could rejuvenate of several molecular characteristics that undergo age-related decline in S cells, including:<br>(i) Heterochromatin foci were re-established in S15

Development • Supplementary information

	For *in vivo* experiments NANOG was expressed locally in the *tibialis anterior* muscle wild-type and LAKIN (*Col1a1*$^{tetO\text{-}Nanog/+}$, *ROSA26*$^{rtTA/rtTA}$, *Lmna*$^{G609G/+}$; *Col1a1*$^{tetO\text{-}Nanog/\ tetO\text{-}Nanog}$, *ROSA26*$^{rtTA/rtTA}$, *Lmna*$^{G609G/+}$; *Col1a1*$^{tetO\text{-}Nanog/+}$, *ROSA26*$^{rtTA/+}$, *Lmna*$^{G609G/+}$ transgenic mice. Local expression was induced by intramuscular injection of a *Dox*-containing hydrogel (Atridox) that enables sustained release of Dox in the surrounding muscle over a period of 3 weeks.<br><br>These workers showed that a single (non-canonical) reprogramming factor, NANOG, could rejuvenate several age-related characteristics *in vivo* and *in vitro*.	cells also significantly decreased nuclear size and increased nuclear circularity.<br><br>*in vivo* experiments showed that NANOG expression in wt animals decreased the percentage of senescence-associated β-galactosidase positive cells amongst *Pax7+* myogenic progenitors; the numbers of myogenic progenitors were also increased in LAKIN mice. In both wt and LAKIN mice the regions that exhibited increased NANOG expression there was increased expression of myosin heavy chain indicating a likely increase in *de novo* myofiber formation.	cells along with the levels of H3K9me3 and H3K27me3, where the latter is likely to be due to the observed transcriptional upregulation of histones and the HMTase-containing PRC2 complex. NANOG expression was required for this restoration because stopping NANOG expression resulted in reversal of the rejuvenating effects of NANOG expression.<br>(ii) NANOG expression reactivates the DNA damage response in S cells.<br>(iii) NANOG expression augments proteolysis and autophagy in S cells.<br>(iv) NANOG expression rejuvenates mitochondrial function.<br><br>Similar results were obtained *in vitro* using isolated myoblasts from *Col1a1*$^{tetO\text{-}Nanog/+}$, *ROSA26*$^{rtTA/+}$, *Lmna*$^{G609G/+}$ LAKI mice. Expression of NANOG in LAKI myoblasts decreased DNA damage, increased mitochondrial function, and cellular energetics as measured by intracellular ATP concentration.
(Alle et al., 2022)	*Dox*-induced expression of OSKM (4F transgene) for two-and-a-half weeks in a two-month old LAKI progeria mouse model. Mice were heterozygous for both the 4F transgene and the LAKI mutation (*Lmna*$^{G606G/+}$).<br><br>The same two-and-a-half week burst of OSKM expression was also induced in non-progeria mice.	The single two-and-a-half week "burst" of OSKM extended life-span of *Lmna*$^{G606G/+}$ mice by 15%. Life-span extension was also observed in non-progeria mice. Also, some 6 months after the transient OSKM expression in progeria mice lean mass was retained, motor skills were enhanced and integrity of bone, lung, spleen, kidney and skin tissues was maintained.	A DNA methylation signature was identified that correlated with the rejuvenation observed in various tissues. The signature was propagated in rejuvenated tissues six months after the two-and-a-half week "burst" of OSKM expression.
(Cheng et al., 2022)	For *in vivo* experiments, cyclical *dox*-inducible expression of four factors OSKM in 4F transgenic mice was induced for 2 days "on" followed by 5 days "off". The effect of cyclical OSKM expression in an intervertebral disc degeneration (IDD) mouse model was investigated. Mice were 8 weeks of age when	*In vivo*, cyclical OSKM treatment 4 weeks after injury (two weeks of cyclical OSKM) showed less histological damage to intervertebral discs compared to controls. This difference was exacerbated 14 weeks after injury (12 weeks after cessation of cyclical OSKM expression).	Dox2d and dox4d treatment of passage 6 senescent NPCs showed down regulation of age-related stress response genes of the p53 pathway, namely p16^{INK4a} p21^{CIP1}, atf3 and ggdd45b. DNA damage as measured by γH2AX was reduced. There was increased expression of anabolism factor col2 and

	IDD was induced by needle puncture into the disc. 2 weeks after injury the cyclical expression of OSKM was administered for 2 weeks.<br><br>The age -related experiments were done *in vitro*. Nucleus pulposus cells (NPCs) were isolated from 4F OSKM transgenic mice and cultured until they has reached replicative senescence (passage 6). OSKM expression was induced senescent cells using different expression paradigms:<br>(i) 2 days “on” (dox2d)<br>(ii) 4 days “on” (dox4d)<br>(iii) 4 days “on” and 4 days “off” (dox4d-4d)<br>(iv) 4 days “on” and 8 days “off” (dox4d-8d)<br>(v) 4 days “on”, 4 days “off” and 4 days “on” (dox4d-4d+4d)	Dox2d and dox4d treatment of passage 6 senescent NPCs showed that by day 4 NPCs took on a more youthful appearance morphologically, *e.g.* decrease in size of nucleus and nucleoli. Cellular identity was not lost by short-term reprogramming as evidenced by the gradual increase in the NPC marker SOX9 over the 4 day induction. SOX 9 is thought to enhance cellularity and organisation of the extracellular matrix in the intervertebral disc.	decrease in the catabolism factors mmp13 and adamts5. The levels of H3K9me3 and H4K20me3 were restored.<br><br>Comparison of reprogramming regimes dox4d-4d, dox4d-8d and dox4d-4d+4d showed that the age reprogramming of age-related characteristics ($p16^{INK4a}$ $p21^{CIP1}$, atf3, ggdd45b, γH2AX, H3K9me3, ROS levels and EdU incorporation) was were reversible in that the dox4d-8d regime saw many of the age-related markers reappearing. Notably the reappearance of the age-related markers were again reversed in the dox4d-4d+4d regime indicating that age reprogramming was short term but could maintained with continued short-term cyclical expression of OSKM.<br><br>Based on the morphological rejuvenation observed in NPCs the effect of OSKM expression on the organisation of the cytoskeleton was investigated. In senescent NPCs F-actin microfilaments take on a disorganised appearance that become more organised after dox4d treatment. As before, dox4d-8d regime saw F-actin microfilaments become dis-organized again, but not with the short-term dox4d-4d regime. Dox4d-4d+4d saw re-organisation of the F-actin microfilaments indicating that the (re-) organisation of F-actin microfilaments was short term and could maintained but continued short-term cyclical expression of OSKM.<br><br>Re-distribution of the cytoskeleton is an energy-driven process. Consistent with this the dox4d-4d+4d regime showed that there was also cyclical rejuvenation of the oxidative phosphorylation pathway, particularly the enzymes of the glycolysis and the TCA pathway. A role for Hexokinase 2 (HK2) was demonstrated as being important for the re-organisation of the cytoskeleton because inhibition of HK2 by 3-Bromopyruvic acid (3-Br PA) also inhibited F-actin organisation in response to OSKM expression.

Development • Supplementary information

			Notably, 3-Br-PA also inhibited the OSKM-driven rejuvenation of other age-related characteristics including senescence-associated β-galactosidase activity and age-related stress response genes of the p53 pathway, namely $p16^{INK4a}$ $p21^{CIP1}$, atf3 and ggdd45b. H4K20me3 levels were not restored after , 3-Br-PA treatment.
(Browder et al., 2022)	Cyclic *dox*-inducible expression of four factors (4F) OSKM with 2 days “on” followed by 5 days “off” in naturally-aged mice using three different treatment paradigms.<br><br>In the first paradigm cyclic expression of OSKM was started at 15 months of age and continued until 22 months of age (7-month treatment, ‘long-term’). Second, cyclic expression was started at 12 months of age and continued until 22 months (10-month treatment, ‘long-term’). The third cyclic expression was started at 25 months of age and continued for 1 month (late onset, ‘short-term’).	Overall health of mice that underwent 7 month, long-term partial reprogramming was unaffected, as evidenced by normal (white and red) blood counts, normal performance in neurological tests (open field and locomotor), and no gross histological changes in treated animals. 7 month long term partial reprogramming was safe.<br><br>7 month long-term partial reprogramming increased the proliferative capacity of epidermal cells which was associated with an increased thickness of the skin compared to untreated mice. In an excisional wound splinting model it was shown that re-epithelisation was enhanced with reduced fibrosis during healing in 7 month long-term partially reprogrammed mice.<br><br>There was no evidence of an increased regenerative potential of muscle after injury (cardiotoxin treatment) in 7 month long-term partially reprogrammed mice, as measured by muscle thickness and PAX7+ve muscle stem cells.	7 month long-term partial reprogramming results in changes in the transcriptome (using RNA-seq) of skin cells showing increased expression of genes involved in lipid and fatty acid metabolism and down-regulation of genes involved in epidermal differentiation. The latter may reflect a less differentiated state of skin cells and could explain the observed increase in proliferative potential of epidermal cells. A similar transcriptome analysis using late onset, 1-month “short-term” mice showed enrichment of genes involved in hair follicle development and the hair cycle, while genes involved in interferon responses were down-regulated.<br><br>Using mice undergoing 10-month long-term partial reprogramming metabolomic and lipidomic analyses of serum samples were undertaken at 17 and 22 months. There were only minor changes after 5-months of treatment. After 10 months major age-related metabolite changes in the serum were reversed.<br><br>DNA methylation analysis revealed that 7 month long-term partial reprogramming could reverse age-related DNA methylation changes in skin and kidney resulting in eAge signatures that resembled younger eAges (up to 0.4 eAge years). Late-onset, short-term reprogramming did not result in eAge changes.
(Chondronasiou et al., 2022)	Used Dox-inducible OSKM transgenic mouse (i4F). OSKM was expressed for one week in 55 week-old and 100 week-old mice.<br><br>The effect on epigenetic, transcriptomic and	After 1 week expression of OSKM histological changes were observed in the pancreas that were reversed two weeks after Dox was stopped. In liver and spleen no histological changes were observed	DNA methylation analysis of the pancreas showed that DNA methylation lost at promoters and enhancers in 55 week-old mice was restored by the end of the 1 week “burst” of OSKM expression – for

Development • Supplementary information

	metabolomic rejuvenation was investigated 2 to 4 weeks after the 1 week "burst" of OSKM expression.	after 1 week expression of OSKM and two weeks recovery.	half of the loci the re-methylation was maintained in the 2 week recovery period. For DNA methylation that was acquired at promoters and enhancers during ageing, demethylation took place only after Dox was stopped. This temporal pattern was not observed in liver.<br><br>Transcriptome analysis of 55 week-old pancreata showed that expression of gene-sets known to change with ageing are rejuvenated 2 weeks after a 1 week "burst" of OSKM expression. These genes set included insulin and mTOR signalling, mitochondrial metabolic processes, and DNA repair.<br><br>In the liver, 5 genes from the mouse aging cell atlas, *Tm9sf2*, *Cd164*, *Ddx5*, *P4hb*, *Lars2*, were shown to be down-regulated in 100 week-old mice and rejuvenated after 1 week expression of OSKM. Two further genes, *Nrf2* and *ApoM* were shown to be down-regulated in 100 week-old mice and rejuvenated by 1 week OSKM expression. The expression of senescence marker, $p16^{INK4a}$ and senesce-associated cytokines Mcp1 and Cxcl2 were not rejuvenated in 100 week-old mice, indicating that 1 week expression of OSKM is not sufficient to rejuvenate the genome of senescent cells in old livers.<br><br>Metabolomic analysis of sera from OSKM-treated 100 week-old mice showed that age-related changes in 4 metabolites (4-hydroxyproline, thymine, trimethyl-lysine and indole-3-propionic acid) were rejuvenated after 1 week expression of OKSM.
(Gill et al., 2022)	Used Dox-inducible OSKM lentiviral vector to transiently express OSKM for 10, 13, 15 or 17 days in human dermal fibroblasts (HDFs) derived from middle-aged donors; chronological ages 38, 53 and 53; eAges according to Horvath clock were 45, 49 and 55. After 10, 13, 15 or 17 days of OSKM expression cells were culture for 4-5 weeks in the absence of Dox.	HDFs that became SSEA+ve and CD13-ve during the 10-17 day transient reprogramming period were termed "transient reprogramming intermediate" and the same cells after the 4-5 week recovery period termed "transiently reprogrammed fibroblasts".<br><br>Elongated HDFs become significantly more "round" upon transition to "transient	Based on a fibroblast-specific gene set (4178 genes) it was shown that during transient reprogramming 2803 genes (cluster 1) showed temporary down-regulation, 961 genes (cluster 2) temporarily up-regulated. Notably, 414 genes (cluster 3) persistently expressed indicating that expression of cluster 3 genes are maintained through transient reprogramming. This persistent expression may

Development • Supplementary information

		reprogramming intermediate" stage and even after 17 days of reprogramming can return to their original elongated morphology when they return back to "transiently reprogrammed fibroblasts".<br><br>HDFs after 13 days reprogramming had increased Collagen I and IV levels that were associated with a weak trend towards increased migration speed in a wound healing assay.	represent transcriptional "memory" through transient reprogramming enabling return to the original starting cellular identity.<br><br>In order to determine the degree of rejuvenation in transiently reprogrammed fibroblasts based on the transcriptomic data two transcription "clocks" were used. The random forest-based clock (median absolute error 12.57 years) calculated reduced mean transcription age by ~ 30years. The BiT transcription clock showed reduced transcriptional age of ~20 years. For both transcription clocks the optimum window for transcriptional rejuvenation were between days 10 to 13 of reprogramming. Any longer (days 15 and 17) the degree of rejuvenation based on these clock was less.<br><br>There was low DNA methylation at fibroblast-specific promoters in all three clusters during transient reprogramming. There was also little change in DNA methylation at the enhancers for all three clusters during transient reprogramming. Notably, there was no DNA hypermethylation at the enhancers and promoters of fibroblast-specific genes in cluster 1 that were down-regulated during transient reprogramming. It was suggested that the persistent hypomethylation in cluster 1 promoters and enhancers might act as the epigenetic "memory" through transient reprogramming that facilities return to the original starting cellular identity.<br><br>The Horvath clock showed that the reduction in median methylation age was ~30 years and the optimum age for methylation rejuvenation was 13 days, similar to what was observed with the transcriptional clock. Reduction in eAge was less after 10 days of reprogramming as well after 15 and 17 days.<br><br>Transient reprogramming on days 10 and 13 was able to reconstitute age-related decline in H3K9me3 levels.

(Roux et al., 2022)	Used Dox-inducible OSKM lentiviral vector to transiently express OSKM for 3 days "on", 3 days "off" regime in aged adipocytes and muscle-derived mesenchymal stem cells (MSCs).<br>The same Dox-inducible lentiviral-based vector was used to drive *Msx1* for 3 day "on" 3-4 days "off" regime in aged myocytes. *Msx1* is known to de-differentiate myocytes into a multi-potent state.	Absent whole animal and cytological studies, single cell transcriptomics was used to characterise partial reprogramming in young and aged cells. Briefly, this study investigated: (i) the relationship between restoration of a youthful transcriptional profile and the suppression of cellular identity, (ii) whether subsets of reprogramming factors are sufficient for transcriptional rejuvenation and, (iii), whether factors that drive multi-potency, rather than pluri-potency, can rejuvenate the transcriptional profile	Single-cell (sc) RNA-Seq analysis was undertaken on 30,000+ adipogenic cell mRNA profiles and 20,000+ MSC profiles. Transcriptomic analyses showed that a young transcriptional profile is restored in old mesenchymal and adipogenic cells after transient expression of OSKM. The effectiveness of the restoration is dependent on cell type and is associated with a transient suppression of cellular identity.<br><br>Screening different reprogramming combinations showed that no single reprogramming factor can restore a youthful transcriptional profile although the oncogenic free *Oct4*/*Sox2* combination restored a youthful transcriptional profile with a reduced effect on the loss of cellular identity, indicating that this combination may minimize the risks of suppressed cellular identity and potential neoplastic transformation.<br><br>The conclusion drawn from the analysis of the reprogramming combinations is that suppression of cell identity and restoration of youthful gene expression and not tightly coupled albeit none of the combinations tested could completely disentangle these two phenotypic characteristics.<br><br>Genes that are targeted by the PRC2 complex (containing the Ezh2/1 H3K27me3 HMTase) are refractory to partial reprogramming. This is to the advantage of age reprogramming because genes targeted by PRC2 are known to be important for retaining cellular identity and it is the aim of age reprogramming to rejuvenate cells without loss of cellular identity.<br><br>Transient expression of *Msx1*can partially restore a youthful transcriptional profile in aged myogenic cells. It would seem that in addition to the classical "reprogramming factors" that drive pluripotency, genes that expand developmental potential (oligopotency) can also rejuvenate old cells.

Development • Supplementary information
